# Effect of *Antrodia camphorata* on Inflammatory Arterial Thrombosis-Mediated Platelet Activation: The Pivotal Role of Protein Kinase C

**DOI:** 10.1155/2014/745802

**Published:** 2014-10-14

**Authors:** Wan-Jung Lu, Shih-Chang Lin, Chang-Chou Lan, Tzu-Yin Lee, Chih-Hsuan Hsia, Yung-Kai Huang, Hsiu-Chuan Lee, Joen-Rong Sheu

**Affiliations:** ^1^Graduate Institute of Medical Sciences and Department of Pharmacology, College of Medicine, Taipei Medical University, 250 Wu-Hsing St., Taipei 11031, Taiwan; ^2^School of Nutrition and Health Sciences, Taipei Medical University, Taipei 11031, Taiwan; ^3^Division of Allergy and Immunology, Department of Internal Medicine, Cathay General Hospital, Taipei 10630, Taiwan; ^4^The Laboratory of Allergy and Immunology, Cathay Medical Research Institute, New Taipei City 22171, Taiwan; ^5^Department of Medicine, School of Medicine, Fu Jen Catholic University, New Taipei City 24205, Taiwan; ^6^Sheen Chain Biotechnology, Co. Ltd., Taipei 11503, Taiwan; ^7^School of Oral Hygiene, College of Oral Medicine, Taipei Medical University, Taipei 11031, Taiwan; ^8^Program for Translation Medicine, Taipei Medical University, 250 Wu-Hsing St., Taipei 11031, Taiwan

## Abstract

*Antrodia camphorata* is a rare Taiwanese medicinal mushroom. *Antrodia camphorata* extract has been reported to exhibit antioxidant, anti-inflammation, antimetastasis, and anticancer activities and plays a role in liver fibrosis, vasorelaxation, and immunomodulation. Critical vascular inflammation leads to vascular dysfunction and cardiovascular diseases, including abdominal aortic aneurysms, hypertension, and atherosclerosis. Platelet activation plays a crucial role in intravascular thrombosis, which is involved in a wide variety of cardiovascular diseases. However, the effect of *Antrodia camphorata* on platelet activation remains unclear. We examined the effects of *Antrodia camphorata* on platelet activation. In the present study, *Antrodia camphorata* treatment (56–224 *μ*g/mL) inhibited platelet aggregation induced by collagen, but not U46619, an analogue of thromboxane A_2_, thrombin, and arachidonic acid. *Antrodia camphorata* inhibited collagen-induced calcium (Ca^2+^) mobilization and phosphorylation of protein kinase C (PKC) and Akt. In addition, *Antrodia camphorata* significantly reduced the aggregation and phosphorylation of PKC in phorbol-12, 13-dibutyrate (PDBu) activated platelets. In conclusion, *Antrodia camphorata* may inhibit platelet activation by inhibiting of Ca^2+^ and PKC cascade and the Akt pathway. Our study suggests that *Antrodia camphorata* may be a potential therapeutic agent for preventing or treating thromboembolic disorders.

## 1. Introduction

*Antrodia camphorata* is a rare Taiwanese medicinal mushroom that is popularly known as “niu cheng zhi” in Taiwan [1].* Antrodia camphorata* has been used in traditional Chinese medicine to treat food poisoning, drug intoxication, diarrhea, abdominal pain, hypertension, skin irritation, and cancer [2]. Studies have identified bioactive compounds of* Antrodia camphorata*, including polysaccharides, maleic/succinic acid derivatives, triterpenoids, benzenoids, and benzoquinone derivatives [3, 4]. In addition,* Antrodia camphorata* was reported to induce apoptosis in SKOV-3 cells through ROS generation, loss of HER-2/neu activation, and suppression of its downstream signaling pathways, including the PI3K/Akt cascade [5]. In addition,* Antrodia camphorata* inhibited lipopolysaccharide- (LPS-) induced NO production in macrophages [6]. Recent studies have reported that* Antrodia camphorata* is involved in various biological activities, including antioxidant, anti-inflammation, antimetastasis, and anticancer activities as well as liver fibrosis, vasorelaxation, and immunomodulation [7–9].

Critical vascular inflammation leads to vascular dysfunction and cardiovascular diseases, including abdominal aortic aneurysms, hypertension, and atherosclerosis. Intravascular thrombosis is involved in a wide variety of cardiovascular diseases (CVDs). Intraluminal thrombosis is believed to be initiated by platelet adherence and aggregation. Thus, in addition to mediating hemostasis, platelet aggregation may play a crucial role in atherothrombotic processes [10].

Blood platelet activation and aggregation constitute a common denominator in atherothrombotic events and various inflammatory diseases. Platelets have been viewed exclusively as mediators of thrombosis and hemostasis, but a study recently indicated that they play key roles in inflammation and immunity [11]. Therefore, the use of antiplatelet agents to treat thromboembolic diseases (myocardial infarction, ischaemic stroke, and vascular death) warrants investigation. During a preliminary study, we observed that* Antrodia camphorate* at 224 *μ*g/mL inhibited the collagen-induced aggregation of washed human platelets. The influence of* Antrodia camphorata* on platelet activation has yet to be investigated thoroughly. We systematically examined the effects of* Antrodia camphorata* on human platelets and characterized the detailed mechanisms of* Antrodia camphorata*-mediated inhibition of platelet activation.

## 2. Materials and Methods

### 2.1. Plant Material

Crude extracts of* Antrodia camphorata* (70%) were provided by Well Shine Biotechnology Development Co., Pvt. Ltd., Taipei, Taiwan.

### 2.2. Materials

Type I collagen and phorbol-12, 13-dibutyrate (PDBu) were purchased from Sigma (St Louis, MO). Fura 2-AM was purchased from Molecular Probe (Eugene, OR). The anti-Akt (pan) (40D4) monoclonal antibody (mAb), anti-phospho-Akt (Ser^473^) polyclonal antibody (pAb), anti-phospho-(Ser) protein kinase C (PKC) substrate pAb, anti-phospho-p38 mitogen-activated protein kinase (MAPK) (Thr^180^/Tyr^182^) pAb, anti-p38 MAPK (5F11) mAb, anti-phospho-p44/42 MAPK (ERK1/2) (Thr^202^/Tyr^204^) pAb, anti-p44/42 MAPK (137F5) mAb, anti-phospho-c-Jun N-terminal kinse (JNK) (Thr^183^/Tyr^185^) mAb, and anti-JNK pAb were purchased from Cell Signaling (Beverly, MA). The anti-*α*-tubulin mouse mAb was purchased from Thermo Scientific (Waltham, MA). The Hybond-P polyvinylidene difluoride (PVDF) membrane, an enhanced chemiluminescence (ECL) western blotting detection reagent, a horseradish-peroxidase (HRP)-conjugated donkey anti-rabbit IgG, and a sheep anti-mouse IgG were purchased from Amersham (Buckinghamshire, UK).

### 2.3. Platelet Aggregation Assay

Our study was approved by the Institutional Review Board of Taipei Medical University and conformed to the directives of the Helsinki Declaration. All human volunteers provided informed consent. Human platelet suspensions were prepared as described in a previous report [10]. Blood was collected from healthy human volunteers who had taken no medication during the preceding 2 weeks, and the blood samples were mixed with acid-citrate-dextrose solution. After centrifugation at 120 g for 10 min, the supernatant (platelet-rich plasma; PRP) was supplemented with PGE_1_ (0.5 *μ*M) and heparin (6.4 IU/mL) and then incubated for 10 min at 37°C and centrifuged at 500 g for 10 min. The platelet pellets were suspended in 5 mL of Tyrode's solution, pH 7.3 [containing (mM) NaCl 11.9, KCl 2.7, MgCl_2_ 2.1, NaH_2_PO_4_ 0.4, NaHCO_3_ 11.9, and glucose 11.1]; then apyrase (1.0 U/mL), PGE_1_ (0.5 *μ*M), and heparin (6.4 IU/mL) were added, and the mixture was incubated for 10 min at 37°C. After centrifugation of the suspensions at 500 g for 10 min, the washing procedure was repeated. The washed platelets were suspended in Tyrode's solution containing 3.5 mg/mL of bovine serum albumin (BSA), and the final Ca^2+^ concentration in the solvent of the suspensions was adjusted to 1 mM.

A Lumi-Aggregometer (Payton Associates, Scarborough, ON, Canada) was used to measure platelet aggregation as described in a previous report [10]. Platelet suspensions (3.6 × 10^8^ cells/mL) were preincubated with* Antrodia camphorata* at various concentrations or a solvent control (0.5% DMSO) for 3 min before agonists were added under a stirring condition. The reaction was allowed to proceed for 6 min, and the extent of aggregation was expressed in light transmission units.

### 2.4. Measurement of Platelet-Relative Ca^2+^ Mobilization by Using Fura 2-AM Fluorescence

After centrifugation of the citrated whole blood at 120 ×g for 10 min, the supernatant was incubated with 5 *μ*M Fura 2-AM for 1 h with constant stirring condition. As described above, human platelets were then prepared. Finally, the external Ca^2+^ concentration of the platelet suspensions was adjusted to 1 mM. The relative Ca^2+^ mobilization was measured using a CAF 110 fluorescence spectrophotometer (Jasco, Tokyo, Japan) at excitation wavelengths of 340 and 380 nm and an emission wavelength of 500 nm as described in a previous report [12].

### 2.5. Immunoblotting

Washed platelets (1.2 × 10^9^ cells/mL) were preincubated with 112, 224, or 448 *μ*g/mL of* Antrodia camphorata* or a solvent control for 3 min, and agonists were added to trigger platelet activation under a stirring condition. After the reaction was stopped, platelets were immediately resuspended in 200 *μ*L of lysis buffer. Samples containing 80 *μ*g of protein were separated on a 12% acrylamide gel by performing sodium dodecyl sulfate polyacrylamide gel electrophoresis (SDS-PAGE), and the proteins were electrotransferred through semidry transfer (Bio-Rad, Hercules, CA). The blots were blocked with TBST (10 mM Tris-base, 100 mM NaCl, and 0.01% Tween 20) containing 5% BSA for 1 h and then probed with various primary antibodies. Membranes were incubated with an HRP-linked anti-mouse IgG, anti-goat IgG, or anti-rabbit IgG (diluted 1 : 3000 in TBST) for 1 h. Immunoreactive bands were detected using an enhanced ECL system. Ratios of the semiquantitative results were obtained by scanning the reactive bands and quantifying the optical density by using a video densitometer and the Biolight, Version V2000.01, computer software (Bioprofil,Vilber Lourmat, France).

### 2.6. Data Analysis

The experimental results are expressed as the mean ± SEM and are accompanied by the number of observations (*n*). Values of *n* refer to the number of experiments, each of which were conducted using different blood donors. The results of the experiments were evaluated using an analysis of variance (ANOVA). When the ANOVA indicated significant differences among the group means, each group was compared using the Student-Newman-Keuls method. The results of comparisons with a *P* value less than 0.05 were considered statistically significant. All statistical analyses were performed using the SAS, Version 9.2 software package (SAS Institute, Cary, NC).

## 3. Results

### 3.1. Effects of* Antrodia camphorata* on Platelet Aggregation and Intracellular Calcium Mobilization

**Figure 1:**
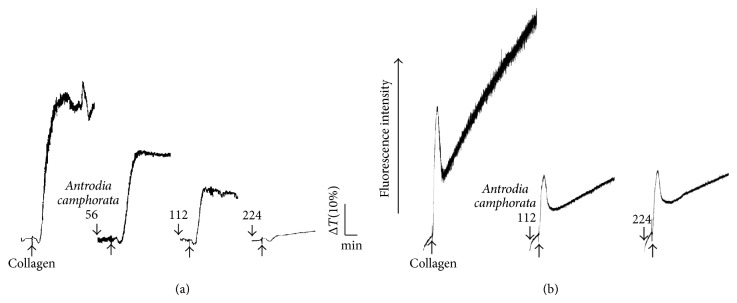
Effects of* Antrodia camphorata* on the regulation of platelet aggregation and calcium mobilization in washed human platelets. Washed platelets (3.6 × 10^8^ cells/mL) were preincubated with a solvent control (DMSO, 0.05%) or 56–224 *μ*g/mL of* Antrodia camphorata*, and 1 *μ*g/mL of collagen was subsequently added to trigger (a) platelet aggregation and (b) relative Ca^2+^ mobilization. Profiles are representative examples of 3 independent experiments.

As shown in Figure 1(a),* Antrodia camphorata* (56–224 *μ*g/mL) inhibited platelet aggregation following treatment with 1 *μ*g/mL of collagen. In subsequent experiments, 1 *μ*g/mL of collagen was used as an agonist to stimulate platelet aggregation. As shown in Figure 1(b), calcium mobilization in human platelets stimulated with 1 *μ*g/mL of collagen was inhibited by 112 or 224 *μ*g/mL of* Antrodia camphorata* in a concentration-dependent manner. However, at a concentration of 448 *μ*g/mL,* Antrodia camphorata* did not significantly inhibit platelet aggregation stimulated by 1 *μ*M U46619, 0.01 U/mL of thrombin, or 60 *μ*M AA (data not shown).

### 3.2. Effects of* Antrodia camphorata* on Mitogen-Activated Protein Kinases Activation

**Figure 2:**
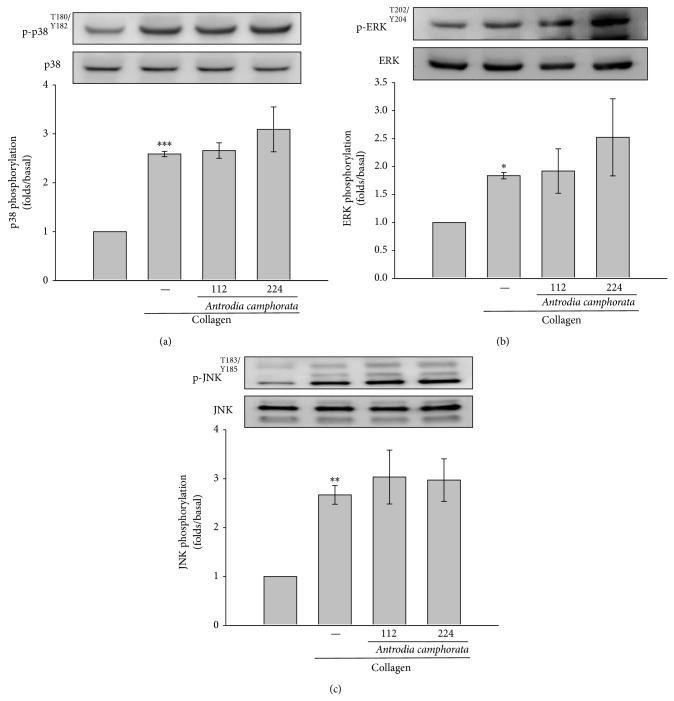
Effects of* Antrodia camphorata* on MAPK activation in collagen-activated platelets. Washed platelets (1.2 × 10^9^ cells/mL) were preincubated with 112 or 224 *μ*g/mL of* Antrodia camphorate* and subsequently treated with 1 *μ*g/mL of collagen to induce platelet activation. The platelets were collected, and the phosphorylation of (a) p38, (b) ERK, or (c) JNK in the subcellular extracts was analyzed. Data are presented as the mean ± SEM (*n* = 3; ^*^ *P* < 0.05, ^**^ *P* < 0.01, and ^***^ *P* < 0.001, compared with solvent control platelets).

The MAPKs control major cellular responses in eukaryotic organisms and contribute to cell proliferation, migration, and differentiation as well as apoptosis.* Antrodia camphorata* did not inhibit collagen-mediated phosphorylation of p38 (Figure 2(a)), ERK (Figure 2(b)), or JNK (Figure 2(c)). These results suggest that* Antrodia camphorata* does not antagonize collagen-mediated MAPKs intracellular signaling events that occur during platelet activation.

### 3.3. Effects of* Antrodia camphorata* on Protein Kinase C Activation

**Figure 3:**
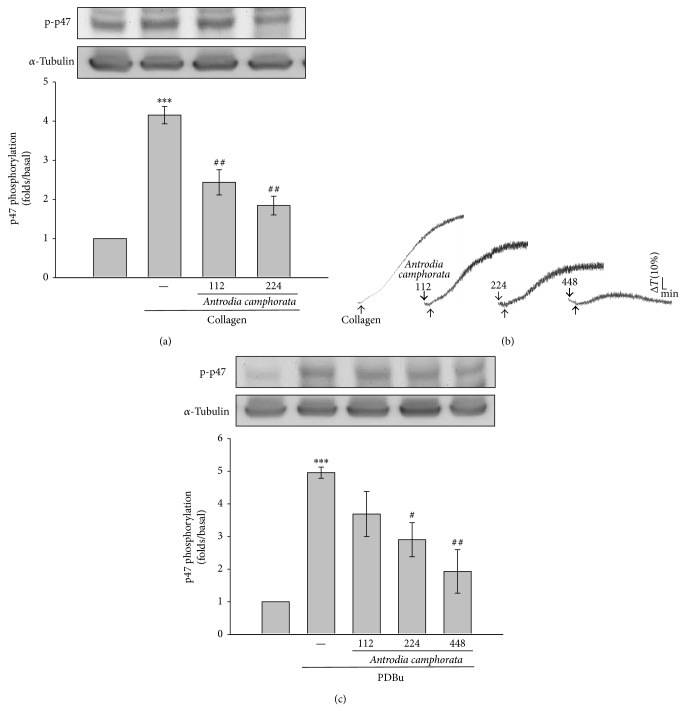
Influence of* Antrodia camphorata* on PKC activation in activated platelets. ((a) and (c)) Washed platelets were preincubated with 112, 224, or 448 *μ*g/mL of* Antrodia camphorata* and subsequently treated with 1 *μ*g/mL of collagen or 150 nM PDBu to induce p47 phosphorylation, the PKC downstream ((a) and (c)), and (b) platelet aggregation. Data are presented as the mean ± SEM (*n* = 3; ^***^ *P* < 0.001 compared with solvent control platelets; ^#^ *P* < 0.01 and ^##^ *P* < 0.01 compared with the collagen group). Profiles (b) are representative of 3 independent experiments.

Activation of platelets by various agonists could lead to the induction of PKC activation and subsequent phosphorylation of p47 proteins [13]. As compared to the protein profile of nonactivated platelets, a protein with an apparent molecular weight similar to that of p47 (47 kDa) was predominately phosphorylated in collagen- (Figure 3(a)) and PDBu- (150 nM; Figures 3(b) and 3(c)) activated human platelets.* Antrodia camphorata* treatments reduced apparent p47 phosphorylation in both collagen- and PDBu-activated platelets (Figures 3(a) and 3(c)). In addition, 448 *μ*g/mL of* Antrodia camphorata* significantly reduced the aggregation of PDBu-activated platelets (Figure 3(b)), indicating that* Antrodia camphorata* directly affects PKC activation in human platelets.

### 3.4. Effects of* Antrodia camphorata* on Akt Activation

**Figure 4:**
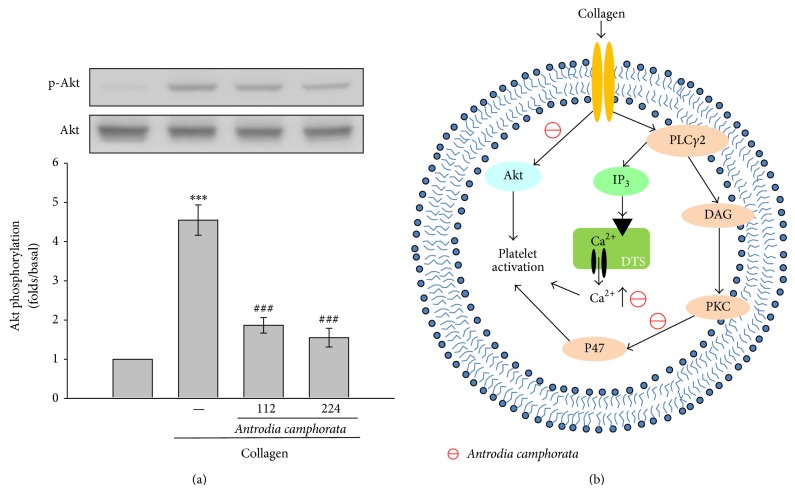
Effects of* Antrodia camphorata* on Akt phosphorylation in collagen-activated platelets. Washed platelets (1.2 × 10^9^ cells/mL) were preincubated with 112 or 224 *μ*g/mL of* Antrodia camphorata* and subsequently treated with 1 *μ*g/mL of collagen to induce platelet activation. The platelets were collected, and the phosphorylation of (a) Akt in the subcellular extracts was analyzed. Data are presented as the mean ± SEM (*n* = 3; ^***^ *P* < 0.001 compared with solvent control platelets; ^###^ *P* < 0.001 compared with the collagen group). (b) Schematic illustration of* Antrodia camphorata*-mediated inhibition of platelet activation. Activated phospholipase C*γ*2 (PLC*γ*2) catalyses the conversion of phosphatidylinositol 4,5-bisphosphate (PI4,5-P_2_) into 1,2-diacylglycerol (DAG) and inositol 1,4,5-trisphosphate (IP_3_). DAG activates protein kinase C (PKC) and, subsequently, phosphorylation of a 47-kDa protein (p47). IP_3 _induces the release of Ca^2+^ from the dense tubular system (DTS).

As shown in Figure 4(a),* Antrodia camphorata* concentration (112 or 224 *μ*g/mL) dependently attenuated Akt phosphorylation stimulated by 1 *μ*g/mL of collagen. In addition,* Antrodia camphorata* did not affect MAPKs phosphorylation in collagen-activated human platelets (Figure 2). These results revealed that* Antrodia camphorata* may prevent collagen-induced platelet activation through the inhibition of PKC and Akt phosphorylation (Figure 4(b)).

## 4. Discussion

This study demonstrated for the first time that* Antrodia camphorata* exhibits potent antiplatelet activity via inhibiting both PKC and Akt activation in washed human platelets (Figure 4(b)).* Antrodia camphorata* has been used in traditional Chinese medicine to treat food poisoning, drug intoxication, diarrhea, abdominal pain, hypertension, skin irritation, and cancer [2]. Recent studies have reported that* Antrodia camphorata* induces substantial apoptosis in human promyelocytic leukemia (HL-60) cells [14]. Another study proved that* Antrodia camphorata* extracts may be used as an adjuvant antitumor agent for human hepatoma cells, which are resistant to most other antitumor agents. Our previous study demonstrated that* Antrodia camphorata* provides effective protection against carbon tetrachloride (CCl_4_) induced hepatic injury in vivo by mediating antioxidative and free radical scavenging activities [15], and it was shown to reduce H_2_O_2_-induced lipid peroxidation and upregulate the expression of hepatic glutathione-dependent enzymes, thereby protecting the rat liver from CCl_4_-induced damage [16].

Phospholipidase (PL) activation may significantly alter by the occurrence of platelets activation by agonists, such as collagen. Inositol 1,4,5-trisphosphate (IP_3_) and 1,2-diacylglycerol (DAG) are produced during the activation of phospholipidase C (PLC), which activates PKC, and subsequently induce the phosphorylation of p47 [17]. It has been proposed that activation of PKC may facilitate certain responses to specific activating signals in distinct cellular compartments [18]. The 6 families of PLC enzymes are found to be categorized: PLC*β*, PLC*γ*, PLC*δ*, PLC*ε*, PLC*ζ*, and PLC*η* [18]. The PLC*γ* family comprises isozymes 1 and 2 and isoform 2 is found to associate in collagen-dependent signaling in platelets [19]. IP_3_ triggers an increase in intracellular Ca^2+^ from Ca^2+^ storage sites (i.e., the dense tubular system, DTS) in platelets. DAG activates PKC-inducing protein phosphorylation (p47) (Figure 4(b)). PKC activation is a strategy adopted by cells to enable certain responses to specific activating signals in distinct cellular compartments [18]. In our study, the activation of both Ca^2+^ and PKC by collagen was diminished in the presence of* Antrodia camphorata*.* Antrodia camphorata* exerted direct effects on PKC activation because it reduced PDBu-induced PKC activation and PDBu-induced platelet aggregation, suggesting that* Antrodia camphorata*-mediated inhibition of platelet activity involves the Ca^2+^ and PKC cascade.

MAPKs include ERKs, p38, and JNKs which are involved in cell proliferation, migration, differentiation, and apoptosis. ERKs, JNKs, and p38 have consistently been identified in platelets [20] and they are activated in platelets stimulated by collagen and thrombin and are involved in thrombosis [21]. ERK and p38 play a vital role in stimulating granule secretion and facilitating clot retraction [22]. In addition, p38 plays a crucial role in activating cytosolic phospholipase A_2_, which produces thromboxane A_2_ by catalyzing AA release [23]. Moreover, JNK1 is reportedly involved in collagen-induced platelet aggregation and thrombus formation [24]. The time of thrombus formation was significantly prolonged in JNK1−/− arterioles in an* in vivo* model and platelet secretion was impaired in JNK1−/− platelets* in vitro* [25]. Akt is a downstream effector of PI3-kinase [26], and previous studies found that Akt-knockout mice exhibited defects in agonist-induced platelet activation [27, 28]. In this study, we demonstrated that* Antrodia camphorata* inhibits the activation of Akt, but not MAPKs, suggesting that the* Antrodia camphorata*-mediated inhibition of platelet activation may involve inhibition of the Akt cell-signaling pathway.

In conclusion, we demonstrated that the antiplatelet activity of* Antrodia camphorata* may inhibit the Ca^2+^ and PKC cascades and Akt signaling pathway (Figure 4(b)). These alterations reduce platelet activity and ultimately inhibit platelet aggregation. Our findings suggest that* Antrodia camphorata* may be a potential therapeutic agent for preventing or treating thromboembolic disorders.
